# Palaeoatmosphere facilitates a gliding transition to powered flight in the Eocene bat, *Onychonycteris finneyi*

**DOI:** 10.1038/s42003-024-06032-9

**Published:** 2024-03-26

**Authors:** Norberto P. Giannini, Alan Cannell, Lucila I. Amador, Nancy B. Simmons

**Affiliations:** 1 Unidad Ejecutora Lillo, CONICET-Fundación Miguel Lillo, Tucumán, Argentina; 2 Facultad de Ciencias Naturales e Instituto Miguel Lillo, Universidad Nacional de Tucumán, Tucumán, Argentina; 3 Department of Mammalogy, Division of Vertebrate Zoology, American Museum of Natural History, NY, USA; 4 ISIPU - Istituto Italiano di Paleontologia Umana, Rome, Italy; 5 Instituto de Estudos Avançados, Universidade de São Paulo, São Paulo, Brasil

**Keywords:** Evolution, Palaeontology

## Abstract

The evolutionary transition to powered flight remains controversial in bats, the only flying mammals. We applied aerodynamic modeling to reconstruct flight in the oldest complete fossil bat, the archaic *Onychonycteris finneyi* from the early Eocene of North America. Results indicate that *Onychonycteris* was capable of both gliding and powered flight either in a standard normodense aerial medium or in the hyperdense atmosphere that we estimate for the Eocene from two independent palaeogeochemical proxies. Aerodynamic continuity across a morphological gradient is further demonstrated by modeled intermediate forms with increasing aspect ratio (AR) produced by digital elongation based on chiropteran developmental data. Here a gliding performance gradient emerged of decreasing sink rate with increasing AR that eventually allowed applying available muscle power to achieve level flight using flapping, which is greatly facilitated in hyperdense air. This gradient strongly supports a gliding (trees-down) transition to powered flight in bats.

## Abstract (web summary)

Aerodynamic modeling based on the primitive, early Eocene bat Onychonycteris strongly supports a gliding transition to powered flight in mammals, which is facilitated under the hyperdense atmospheric conditions here inferred for the fossil age.

## Introduction

**Fig. 1:**
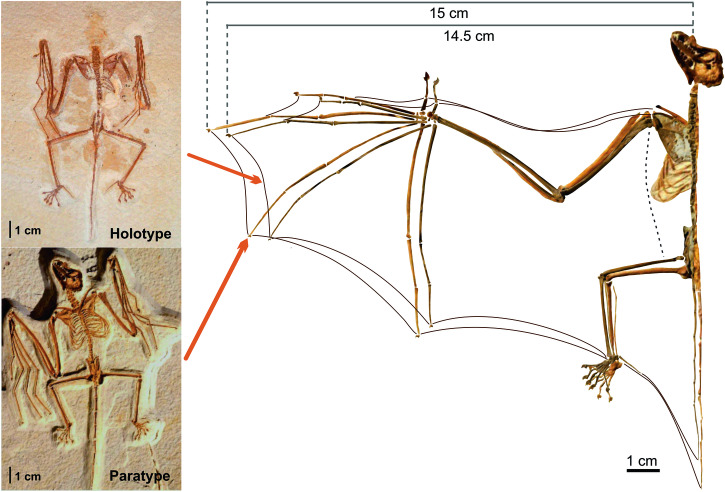
Reconstructed aerofoil of the two existing *Onychonycteris finneyi* specimens. Half wingspan is indicated on top. Insets: dorsal view of holotype ROM 55351 A, ventral view of paratype AMNH 142467, and selected parameter values.

Powered flight is the most demanding mode of animal locomotion, and the three independent origins of powered flight in vertebrates are among the major macroevolutionary transitions of the Phanerozoic^1^. These events were widely spaced in geologic time: pterosaurs first evolved powered flight in the Late Triassic^2^; dinosaurs (birds) in the Late Jurassic^1^; and mammals (bats) in the Early Eocene^3^. Our understanding of the origins of bat flight is precarious. First advanced by Darwin^4^ in his 1859 *Origin of Species*, the current gliding hypothesis of bat flight evolution rests on the transformation of the hand into a webbed and elongated handwing integrated into a preexisting glider *bauplan*^5^. Gliding is thought to have evolved independently at least seven times in mammals^6^. These gliders possess up to three separate skin membranes between the body and legs, the pro-, plagio- and uropatagium^7^, that together act as an aerofoil; these are all present in bats, plus the handwing, or dactylopatagium (Fig. 1). The dactylopatagium is a retention into adulthood of the embryonic interdigital tissue of the hand primordium or handplate, a developmental process controlled by a regulatory circuit involving expression of the genes *Bmp2*, *Gre*, *Fgf8,* and *Shh* that prevents interdigital apoptosis (programmed cell death) caused by *Bmp2* that otherwise produces separate digits in terrestrial mammals^8^. In addition, the characteristic digital elongation seen in the developing bat handwing involves *Bmp2* gene expression within epiphyseal cartilage upregulated by c. 30% as compared to a mouse model, which keeps fingers 2-to-5 growing^9^. Remarkably, bat feet are neither webbed nor elongated, so this regulatory configuration is unique to the hand, as well as to bats as a group, and constitutes the developmental foundation of the bat handwing evolution^10,11^.

In birds, gliding is a derived locomotion mode, typically coupled with some form of soaring or intermittent flight^12,13^; so all gliding birds can also fly, and all possess mid-to-high-AR wings that operate at low angles of attack^12,13^. By contrast, gliding mammals lack a major handwing contribution to the aerofoil, thereby operating low-aspect-ratio wings (AR ≤ 2) at steep angles of attack^14^, while extant bats fly high-AR (≥6) wings capable of low-angle-of-attack performance^15^. Thus, unlike birds, obligate gliding and powered flight seem aerodynamically divergent in mammals, each locomotion mode with its own set of optimal aerodynamic parameters^14^ such that a wide morpho-functional gap exists^16^ between powered fliers (just bats) and all extant^7^ or fossil^6^ mammalian gliders. Alternative hypotheses of bat flight evolution have rejected climbing-and-gliding intermediates (trees-down) and chiefly favor a vertical (ground-up) take-off scenario^17–19^. However, the latter has been questioned on aerodynamic grounds as it requires the initial capability of a particularly demanding mode of flight, both in terms of power and kinematic complexity^20^. Thus, the origin of bat flight remains obscure, with currently no theory satisfactorily explaining its early evolution.

While extant bats exhibit spectacular adaptations to flight^5,21^, truly intermediate forms are lacking in the fossil record^3^. The oldest fossil bats date from the early Eocene, and key among these forms is *Onychonycteris finneyi*, a 52.5 Ma old North American species known from two complete skeletons^3^. *Onychonycteris* was a small mammal (estimated mean 40 g)^22^, but still larger than most modern bats with median at 12 g^16^. Although its morphology suggests that it was capable of powered flight^3^, this hypothesis has yet to be tested, which is of considerable importance given that the postcranium of *Onychonycteris*, and hence the skeletal frame of its flying apparatus, is less derived than that of any other known bat, living or fossil^3^. Our aim is to investigate the flight performance of *Onychonycteris* and model intermediate forms under the conditions that we infer here for the Eocene atmosphere in order to establish the mechanisms involved in the evolution of powered flight in mammals.

Evolution of flapping flight in diverse organisms may have generally occurred in hyperdense palaeoatmosphere^23^. This may have been the case also in bats, and other biological phenomena, such as the appearance of giant soaring pelagornithid birds^24^, also point to the presence of a denser flight medium during the critical initial phase of bat flight evolution—the early Eocene^3^. We tested the possible hyperdense atmospheric conditions of the Eocene^23^ by means of two independent palaeogeochemical proxies, reconciliation of marine^25^ vs. terrestrial^26^ *p*CO_2_ decoupling, and fractionation of Carbon isotopes^27^ in fossil amber^28^. Then, we took a specimen-based approach to investigate aerodynamics of the reconstructed *Onychonycteris* and intermediate models based on its anatomy, in both normodense and estimated hyperdense atmosphere applying the well-established program Flight v. 1.25^13^, an aerodynamic performance program successfully used with a number of reconstructed fossil taxa^1,29^. We demonstrate flapping *and* gliding capabilities in *Onychonycteris*, and confirm aerodynamic continuity, and hence evolvability, between gliding and flapping in intermediate models, thereby strongly supporting a gliding transition to flapping flight in bats, which is especially likely under the inferred hyperdense atmosphere.

## Results

### Flight in normodense versus hyperdense conditions

Our simulations using Flight 1.25^13^, first run with normodense air, i.e., at standard 1.225 kg m^−^^3^ or atmospheric pressure (PATM) of 1 bar (=100 kPa), yielded a climb rate (vertical component of airspeed) of +0.27 m s^−1^ (Supplementary Table 1). A climb rate ≥0 indicates that *Onychonycteris*, as reconstructed here, was capable of sustaining level flight with available muscle power^13^. However, flight costs (Supplementary Table 1) were high: Power was 46% higher than in extant bats of comparable mass^15^, with myofibril (muscular) work and wingbeat frequency also relatively high (Supplementary Table 1). In addition, flight conditions were dangerous in terms of collision risk: cruising speed was 22% higher (Supplementary Table 1) than horizontal velocities seen in extant bats in a body mass range widely inclusive (mean 4.98 m s^−1^ in 30–300 g pteropodid bats^30^) of that estimated for *Onychonycteris*.

Both approaches used to estimate air density in the early-middle Eocene (see Methods) converge on a PATM with a maximum upper constraint of c. 1.6 bar (=160 kPa), i.e., hyperdense atmosphere. Flight 1.25^13^ was then set to calculate flight parameters with air density equivalent to this PATM. Fast flight becomes more difficult in a hyperdense flight medium, but crucially, flight costs substantially decreased and climb rate greatly improved, making flight remarkably less demanding and also safer with regard to collision risk (which increases greatly with airspeed >6 m s^−1^; see Methods and Supplementary Note 1). Specifically, flight parameters approached values calculated for modern bats flying in normodense medium, including a power decrease of 22% to 0.36 W, airspeed decrease of 28%, and a climb rate increase of 26% (Supplementary Table 1).

In parallel, a sensitivity test using a penalized heavier model with an extra 10% weight (at 44 g) exhibited generally poorer flight parameters: power was an additional 17% higher, myofibrils work and wingbeat frequency higher, while climb rate was lower. However, this heavily penalized model also greatly improved performance under hyperdense conditions (Supplementary Table 1).

### Intermediate models

Investigating flight performance with models has been successfully applied in a variety of fossils^1,29^, as well as in a wide range of situations where animal structure is unconventional from an aerodynamic perspective, as in five-winged feathered dromaeosaurids^31^, membrane-winged scansoriopterygid theropods^29^, giant pelagornithid birds^32^, flying fish^33^. Here we modeled intermediate forms based on the observation that bodily proportions of *Onychonycteris* depart from those seen in terrestrial mammals^3^ but are comparable to those of specialized gliding mammals^16^. Thus, we were able to preserve the overall anatomical structure of *Onychonycteris* while varying the contribution of the handwing to the aerofoil in terms of wing area and AR, which is in line with the requirement for testing the current gliding theory of bat flight origins. We considered four cases of handwing elongation beginning with no elongation and wingspan of 0.16 m (Model 1) and progressing (to Model 4) with elongated digits and wingspan at 0.24 m (see Methods and Supplementary Fig. 1).

Simulations with Models 1 and 2 did not indicate any thrust or weight-supporting lift produced by flapping; Model 3 produced little effective lift but required myofibrils muscle work that exceeded a theoretical maximum^13^ of 57 J kg^-1^, so flapping flight was thus deemed unattainable. Model 4 did respond producing useful aerodynamic forces within theoretical energy bounds in the flapping simulations, suggesting that a mechanical threshold is surmounted at about this wingspan (0.24 m) or AR (at 3.9) for this morphology; still, flight costs and risks associated to airspeed (see Methods and Supplementary Note 1) were all high and it did not achieve level flight in normodense conditions with climb rate at −0.36 m s^−1^ (Supplementary Table 1). Flight parameters of Model 4 greatly improved, however, under hyperdense atmosphere, reducing power requirement, myofibrils work, flight speed, and wingbeat frequency, while almost achieving level flight with climb rate at −0.06 m s^−1^ (Supplementary Table 1). Thus, hyperdense conditions would have allowed sustained flapping flight with a lower power requirement for a model with AR at 3.9, effectively an intermediate value between mammalian gliders (maximum AR at 2.15) and *Onychonycteris* (AR at 5.14).

**Fig. 2:**
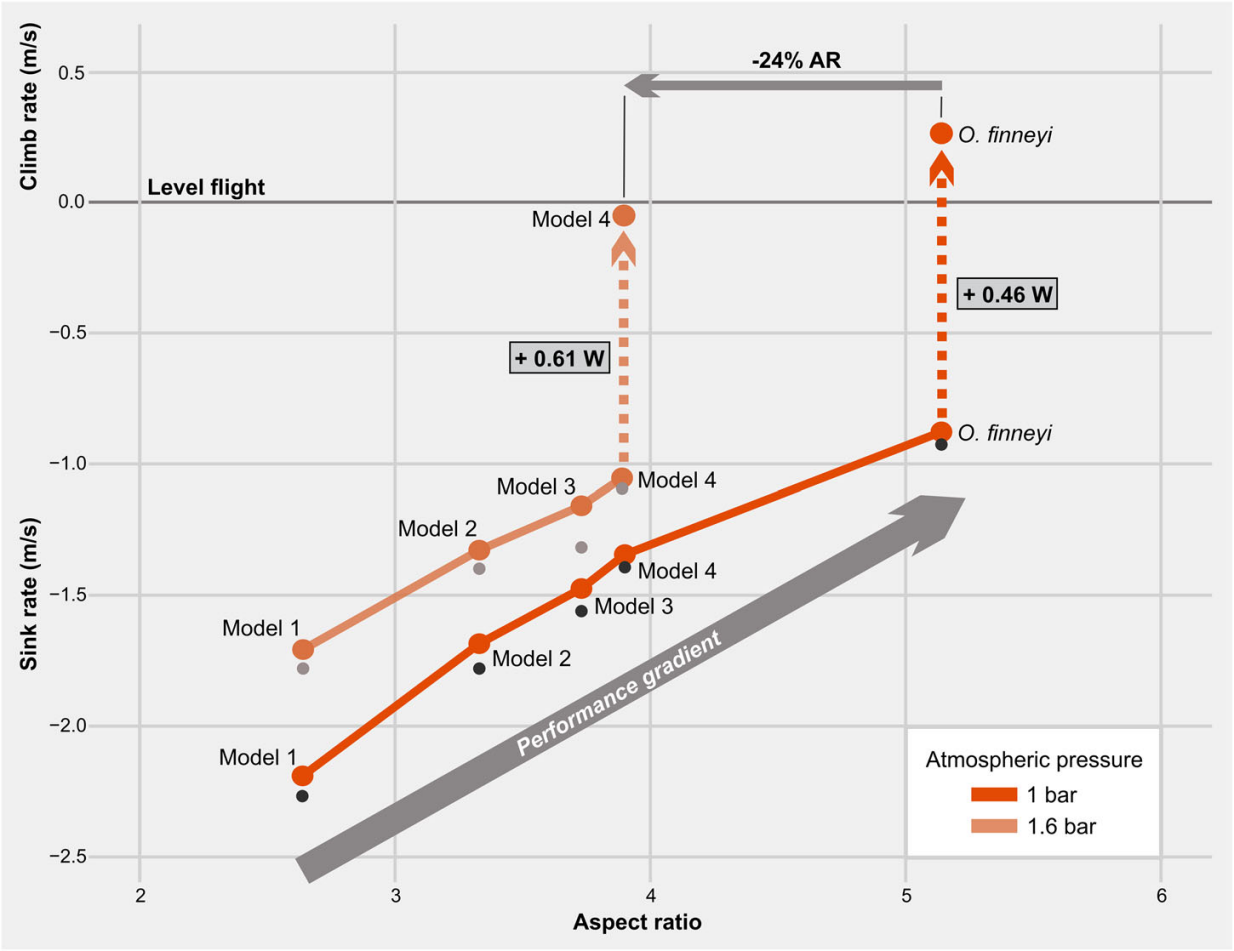
Performance space for the gliding to flapping transition in bats. Positive (climb rate) or negative (sink rate) variation in vertical velocities as function of aspect ratio. Level flight is achieved at 0 m s^−1^ vertical speed^13^. Intermediate models with varying aspect ratio (AR) are indicated as Model 1-to-4. The full-winged fossil is indicated as *O. finneyi*. For the 40 g analysis (see text), variation is shown by dots representing models joined by a full line of increasing AR and decreasing sink rate under normodense (1.0 bar) and hyperdense conditions (1.6 bar). For the 44 g analysis (see text), variation is shown by black (normodense) or gray dots (hyperdense). Decreasing sink rate as AR increases depicts a positive performance gradient (gray arrow) up to the point in which a model responds to flapping, achieving level flight (dotted arrows). This scenario is intermediate between gliding (AR ≤ 2) and flapping (AR ≥ 5) regimes. Numerical data source for this figure in Supplemental Table 7.

With a greater span, as progressing from Model 4 to the full-winged fossil, the flapping frequency (and / or amplitude) decreased, which reduces power, thereby continuing a directional and positive performance gradient toward higher flight efficiency (Fig. 2). In a hyperdense atmosphere, the flapping frequency and hence power requirements decrease further. Specifically, in normodense air wingbeat frequency reduced from 6.9 to 5.3 Hz with increasing AR (from Model 4 to-fossil); in hyperdense air this reduction was 5.8 to 4.4 Hz (see Supplementary Table 1).

### Gliding

We also used Flight 1.25^13^ to simulate gliding performance for all the models outlined above and detailed in Methods (see below), including the full-winged reconstruction of *Onychonycteris*. Gliding starts after sufficient speed is gained from gravity, with glides long enough to disregard losses due to the initial drop; i.e., standard steady-state gliding following a climbing phase to gain height and store potential energy to be released to the air during gliding^15^. Vertical climbing in trees in these models was deemed possible given the small body size and the sharp claws of the *Onychonycteris* hand^3^.

Under normodense conditions, gliding generally improved as AR increased across the models: glide ratio and maneuverability increased, and collision risk was reduced through a decreasing best-glide speed (Supplementary Table 3). Moreover, sink rate (negative vertical component of velocity) decreased with increased AR and wing area, essentially because greater AR reduces induced drag (the drag component incurred by the wingtips), thereby improving the overall lift-to-drag ratio, with the actual full-winged fossil performing better at gliding than any of the intermediate models (Fig. 2). Specifically, gliding performance improved with wingspan from the AR 2.6 Model 1 sinking at −2.19 m s^−1^, to the AR 5.14 full-winged fossil model sinking at −0.88 m s^−1^.

Combining the vertical velocities of sink rate from gliding and climb rate from flapping in a single frame of comparison (Fig. 2), we show that muscle power of the full-winged model was already available (to allow for the observed positive climb rate) to overcome sink by switching to flapping mode (vertical dotted arrow in Fig. 2), thereby using about 3 seconds of flapping to recover the sink from each second spent gliding. These combined results are consistent with a gliding transition to flapping flight under normodense conditions.

Under hyperdense conditions, sink rates and airspeeds decreased for all models tested, making gliding safer (slower) and more maneuverable in terms of tighter turning ability (Supplementary Table 2). Moreover, the muscle power needed to switch from gliding to flapping was also available in Model 4, with climb rate at the verge of sustaining level flight (−0.06 m s^−1^). This key result in the estimated hyperdense medium makes the morphological transition from gliding to flapping shorter by −24% in AR in comparison to *Onychonycteris* flying in normodense air (Fig. 2), allowing for an earlier functional transition to powered flight in a denser Eocene atmosphere. Flapping in Model 4 was highly costly at 0.61 W but still within the theoretical limit of myofibrils work (51.5 J kg^−1^ at minimum power speed), but 20% less costly than flying this model at 1 bar (0.77 W; see Supplementary Table 1).

## Discussion

Our reconstructions illuminate the origins of mammalian flight. We show that the key fossil bat *Onychonycteris finneyi* was capable of both gliding *and* flapping, whereas modeled intermediate forms demonstrate a gliding performance gradient of decreasing sink rate with increasing AR; eventually, flapping becomes viable with available muscle power to sustain level flight (Fig. 2). This continuity supports the current gliding hypothesis of mammalian flight origins on aerodynamic grounds.

Under normodense conditions, gliding must be employed until the model handwing extends the aerofoil to AR ≈ 5 for an ancestral bat similar in size and anatomy to *Onychonycteris*, placing the actual fossil at the very beginning of flapping flight. But this morpho-functional transition is greatly facilitated in a denser flight medium, as level-powered flight is within reach in Model 4 with AR as low as 3.9 at the estimated maximum of 1.6 bar in the Eocene atmosphere, implying an earlier evolutionary switch to a flapping regime (Fig. 2). This latter scenario of an earlier transition of a more primitive form is more likely also from the perspective of clade age, as bat origins have been dated 61.5–57.4 Ma^34^ or slightly older^35^, while the actual age of *Onychonycteris* is younger at 52.5 Ma^3^. Continuing the transformation to increase AR to ≥5 (a Model 4-to-fossil transition) decreases wingbeat frequency and most importantly, cuts power requirements by almost half (Supplementary Table 1), attesting to the potential of AR as a directional evolutionary driver of bat wings.

The adequacy of *Onychonycteris* as model for the gliding transition is easily seen in several features of gliding mammals that are present in *Onychonycteris*. These include joints rotated such that limbs extend laterally in aerial locomotion^6^; the relatively long limbs^3^ that characterize all gliders despite other anatomical differences^36^; and particularly, limb segment differences that suggest distal elongation of the arm, as measured by the dimensionless brachial index (BI: radius-to-humerus length ratio). In *Onychonycteris*, BI is 1.25–1.30^3^, whereas in most gliding mammals BI varies from 0.88 to 1.29, extending into the range of 1.36–1.43 in colugos^6^, which arguably are the most capable mammalian gliders^7^. Therefore, limb structure in *Onychonycteris* is comparable to that of specialized mammalian gliders, making this fossil an appropriate glider model with the handwing removed, as in our Model 1. The adequacy of our model is further reflected in principal parameters like the calculated glide ratio of 2.93 in Model 1 (Supplementary Table 3), which is extremely close to the glide ratio of 2.85 observed in some extant gliders such as the sciurid flying squirrel *Petaurista*^37^.

Remarkably, the performance gradient shown here between gliding and flapping depends only on extending the handwing, an evolutionary transformation strongly supported by developmental data. Rapid evolution of increasing wingspan in bats, and hence AR and concomitant gliding and flapping capabilities, is suggested by a unique molecular regulatory circuit that determines the elongation of a webbed handwing^10,11,38^. This is a key factor in the evolution of mammalian powered flight as the gliding theory on the origin of bat flight requires the evolutionary addition of the handwing to a fully functional gliding *bauplan*^5^.

Our modeling not only supports a gliding transition to flapping flight in paleogene bats; it also compromises key aspects of alternative hypotheses, particularly direct take-off (ground-up) or parachuting proposals (see Introduction). Adding to the fact that the limb structure of reconstructed forms resembles that of gliders, so alternatives like a cursorial scenario become improbable, we have shown here that no useful lift to sustain level flight by means of flapping is produced unless a relatively well-developed handwing is present in addition to patagial tracts extended between body side and legs. Thus, the handwing alone is not enough, given that a handgliding parachuting model with only distal-arm dactylopatagium and lacking other wing membranes^39^ has been demonstrated inferior to a standard gliding *bauplan*^40,41^. Therefore, our models demonstrated gliding *and* flapping capabilities in intermediate forms, and the performance gradient that they show, stands out as strongly suggestive of the more likely transition leading to powered flight in bats.

Theoretical work^15,20,40^ as well as more recent experimental^42^ and robotic^43^ approaches, suggest that low-amplitude wing oscillations aid gliding performance, for instance, by improving lift-to-drag ratio, although these models were based solely on aerodynamic principles, and estimated under standard PATM. Here we quantitatively demonstrate a gliding transition based on the actual features of a key fossil bat with the least derived flight apparatus^3^, in the estimated hyperdense atmospheric conditions of its actual geologic time of occurrence. We suggest that evolutionary responses to high atmospheric density may have been key in the evolution of powered flight^13,23^, as has been suggested for Permian griffinflies (Protodonata)^44^, early Mesozoic^45^ and gigantic Miocene^46^ birds, and as shown here for Eocene bats.

Generally, flapping flight is a complex specialization only attained by a few lineages; besides the atmospheric effects considered here, biomechanical and physiological considerations^15^ suggest that arboreality may be an optimal environment for controlled flight to evolve^47^. The environment in which *Onychonycteris finneyi* lived, as documented in the extraordinarily rich fossil community of the Green River Formation, agrees with this scenario^3^. Flight capacity appears to have originated convergently in at least two distinct paravian lineages, first in birds 150 Ma ago and later in microraptorines between 130 and 120 Ma ago, and in both there is a pattern of proportionally larger wings appearing early in their ontogeny, indicating that the traditional focus on power (i.e., large pectoral muscles) as prerequisites for flight may be incorrect^48^. Thus, paravian and bird flight may also have evolved in conditions where power was less critical^48^, which is more likely in a denser flight medium^23^.

As originally proposed by Darwin^4^, and advanced furthermore recently^5,40,49–51^, the hypothesis of a gliding origin of bat flight as evaluated here most likely represents a case of incremental evolution through intermediate forms of changing function, which may be critical in functionally demanding transitions like the evolution of powered flight in vertebrates^1^. These transitions may be greatly facilitated by favorable extrinsic conditions, such as the palaeoatmospheric densities that we infer for the period of evolution of the unique bat handwing. While the origin of pterosaur flight remains obscure, although it may be related to scansorial habits^2^, a gliding transition to flight in bats stands in striking contrast with the cursorial-dominated transition inferred for birds^1,52^. This highlights the fact that disparate mechanisms and diverse macroevolutionary pathways have been traversed by vertebrates for the conquest of the aerial medium.

## Methods

### Species and specimens

*Onychonycteris finneyi* (Chiroptera: Onychonycteridae) is a fossil bat species known from two nearly complete skeletons from the Fossil Butte Member of the Green River Formation in Wyoming (~52.5 Ma^3^). Phylogenetic analyses, including both extant species and other Eocene fossil bats indicated that *Onychonycteris* is on a basal branch of archaic bats^53^. *Onychonycteris* represents the most primitive of all known bats, retaining relatively plesiomorphic limb proportions as well as claws on all forelimb digits, unlike any other fossil or living bats^3^. This bat species also exhibits relatively primitive features of the ear region, suggesting that it was not capable of echolocation^3^. We examined and measured both the holotype (Royal Ontario Museum ROM 55351 A) and paratype (American Museum of Natural History AMNH 142467) of *Onychonycteris finneyi* to reconstruct body mass and patagial proportions. The two specimens are extraordinarily similar in both bodily proportions (Fig. 1) and weight estimations, having a body mass range of 38–41 g^22^, total aerofoil surface range 0.016–0.018 m^2^, wing loading (WL) range 21.5–24.0 Pa, wingspan of 0.28–0.32 m, and aspect ratio (AR) range of 4.75–5.32^16^. Other important available wing data known for the specimens, such as tip shape index (see ref. ^16^), are not directly used in the aerodynamic model (see below).

### Aerofoil reconstruction

The skeletal frame of the wing was reconstructed by extending the articulated arm and placing the wrist level with the shoulder, keeping the elbow joint flexed at 90° between the humerus and the proximal half of the radius shaft, as is standard^54^ for registering wing form in extant bats. Digits were extended following the anatomical configuration of their joints, which are similar to that of modern bats^3,16^; the vertebral column was straightened, and the hind leg was stretched following joint morphology. Patagia were inserted in that skeletal frame of stretched arms and legs, and were reconstructed as seen in modern bats including a uropatagium (tail membrane) extended from tip of calcar as preserved in the holotype^3^ to tip of tail (Supplementary Fig. 1).

In addition, we constructed models based on the anatomical structure observed in *Onychonycteris* by changing the contribution of the handwing to the aerofoil. Flapping flight and gliding were simulated by changing the extension of the handwing as supported by developmental data^9–11,38,55–62^, first with the handwing reduced to a minimum, i.e., with no handwing contribution to the aerofoil, called Model 1. Such form is similar to gliding mammals with free digits such as the rodent species in the genus *Glaucomys* (flying squirrels; Sciuridae), and diprotodontian marsupials in the genus *Petaurus* (sugar gliders; Petauridae)^7^, but maintaining limb proportions as in the fossil. This is justified because bats are the most preeminent forelimb-dominated mammals^3^ and this is known to be controlled by differential gene expression between embryonic hand- and footplates, making the former larger and developmentally more advanced relative to the latter, differences that are maintained along all development stages^9^. A subsequent form, Model 2, was modeled on the same skeletal frame but assuming a hand with slightly elongated fingers enclosed in the patagium; colugos (extant dermopterans in the genera *Cynocephalus* and *Galeopterus*; Cynocephalidae) exhibit this feature^7^. Wingspan was thus increased to about 20 cm with an estimated AR of 3.3 (Supplementary Fig. 1). Further digit extensions that increase each wing/forelimb and dactylopatagium (=handwing) length by 1 cm per side (i.e. previous wingspan + 2 cm to yield 0.22 m and AR 3.73), termed Model 3, and then again by another 1 cm per side (previous wingspan +2 cm to yield 0.24 m with AR 3.9), termed Model 4. These changes gave the outlines shown in Supplementary Fig. 1, with extended forearm, legs stretched as in gliding mammals (see ref. ^7^), and with a tail membrane as reconstructed in the fossil^3,16^. In these reconstructions, all bodily proportions, with the exception of digits, were held constant as for the full-winged fossil. The basic aerodynamic parameters of wing area, wingspan and aspect ratio are given in Supplementary Table 3 for all four models and the fossil.

### Flight parameters

Bat flight can be exceedingly complex in both natural and experimental conditions^63^, but the simulation model Flight 1.25^13^ used can be applied to bats^13^ to model steady, level, non-accelerating, forward flight, a baseline to establish whether the fossil *Onychonycteris finneyi* and modeled forms are capable of aerial locomotion using flapping with available muscle power. For this purpose, this aerodynamic model^13^ renders acceptable power output in a range of forward airspeeds as compared to actual measures of kinematic energy contained in the wake of bats flying in a controlled setting^64^. These experimental (wind tunnel) conditions cannot be directly applied to fossils, but we used the tested theoretical model^13^ to accurately calculate parameters with variables mensurable in fossils, as in a number of previous studies focusing on other extinct taxa (e.g. refs. ^1,29,32^).

The basic model input data were taken as averages between the two *Onychonycteris* specimens (Fig. 1). The model setup uses input data with no pay-load (i.e., no prey or fetus being carried) and a body drag coefficient of 0.25, based on the values of extant bats of similar mass^65^. The frontal area factor (which considers the aerodynamic form of the head and for most birds is taken as unity) is here increased to 1.3 (see ref. ^65^). The flight muscle fraction was found to be, on average 9.13% of total body mass in extant bats, with a minimum value of 7.8%^66^. Thus, a low (high penalty) value of 8% has been adopted, which takes into account the extra hind limb mass (not used for flight) and robust nature of *Onychonycteris finneyi*^3^; this is lower than the 10% value used for an evaluation of basal gliding or flying theropod dinosaurs^29^. To give a wingbeat similar to medium-sized extant bats, a factor of 0.5 is used, which results in wingbeat of >6 Hz at 1 bar PATM, a value in agreement with the scaling relationship of wingbeat and mass for extant bats^66^, which show a reduction factor of 0.68 in relation to birds^13^; however, with this higher factor the model tends to overestimate wingbeat frequency (7–8 Hz) in relation to extant values as the wing length is shorter in *Onychonycteris* and the models tested. With these specifications, Flight 1.25^13^ was set to calculate power curves and the flapping flight parameters of mechanical power (W), maximum rate of climb (m s^−1^), specific work in myofibrils (J kg^-1^), wingbeat frequency (Hz), minimum power speed (m s^−1^), and maximum range speed (m s^−1^).

Given the relatively longer legs and more robust and archaic nature of the fossils^3^, a sensitivity test was made by increasing the average estimated mass of 40 g by 10% to 44 g. Note that this represents fourfold the actual mass variation of 2.5% seen in fossils, so this heavier model imposes a major penalty on the potential ability to generate lift and thrust. Flapping flight simulations were thus carried out independently for both mass values (40 and 44 g) and for varying air densities, from 1 bar (present low altitude) to a maximum constraint of 1.6 bar estimated to be the air pressure during the early Eocene (see below). We report here the results from fitting models at contrasting 1 bar and 1.6 bar, given that the intermediate results are linear with increasing PATM.

### Glide parameters

The Glide Polar feature of Flight 1.25^13^ can reproduce the glide performance of extant mammals, as well as large soaring birds and man-made ultra-light gliders^13^. The same input data as above were used to calculate Glide Polars with the output parameters of Glide ratio, best-glide speed (m s^−1^), sink rate (m s^−1^), impact speed (m s^−1^), and turn radius (m, at 24° bank). A higher than-default Lift Coefficient (L) of 3 was introduced, to reflect the high camber of the mammalian wing membrane^14,15,20,67^, and considering that the head generates less drag and lift at airspeeds of around 5 m s^−1^.

Mammalian gliding differs from bird gliding in that the aerofoil has a very low AR and operates at a high angle of attack^7,68^. Thus, the dimensionless wing profile drag coefficient adopted by the model has to be modified to account for these characteristics. To adjust this, the following sources were considered. The airfoil NACA0012, utilized in applications such as mini-unmanned aerial vehicles (UAVs), flies at low velocity and at small scales in a low Reynolds number regime; wind tunnel experiments at 13.1 m s^−1^ and air density of 1.225 kg m^-3^ (normodense) showed that the drag coefficient jumped sharply from about 0.1 to 0.2 at an attack angle of 20 degrees, reaching about 0.3 for angles of attack of about 30 degrees in which lift was highest^69^. Similar results were found for a UAV low-speed wing with functional constraints, with the wing drag coefficient going from 0.1 at an angle of attack of 14 degrees (Glide Ratio, GR > 4) to 0.2 at 20 degrees (GR < 2.75)^70^. Also, for a high-AR, bat-inspired membrane wing profile, the wing drag coefficient was not dependent on flapping frequency or AR (2.5 to 4.5), but did depend on amplitude angle (sweep and flap)^71^. With little wing movement, this coefficient was found to be about 0.24 for both the downstroke and upstroke. As the angle of attack for mammalian gliders has been found to be greater than 40 degrees^14^, this suggests that the wing profile drag coefficient would be at least 0.2. This value reproduces squirrel flight and was used in the initial simulation model tests (see below).

Most organisms can withstand impacts at 4.4 m s^−1^ but impacts at greater speeds can be fatal^72^. Average woodland speeds of bats were found^66^ to be 4.8 m s^−1^, and maximum modeled speed of Rhinolophidae^65^ was taken as 6 m s^−1^ (see additional details in Supplementary Information). In our simulations, this speed was thus taken as a practical limiting constraint. Better gliding performance can be obtained by lowering the angle of attack, but this increases airspeed above this limit. However, for the larger AR models in higher density air, it was possible to simulate gliding with a lower angle of attack (to less than 20 degrees) with a Wing Drag Coefficient of 0.1 and still within airspeed limits.

### Aerodynamic model validation

We validated the aerodynamic model by comparing empirical parameters measured in both powered-flying bats and gliding mammals, with the respective output of the model. A mammal glide setup, based on the Indian giant flying squirrel *Petaurista philippensis* (Sciuridae), gave an airspeed of 5.6–8.6 m s^−1^ and a glide ratio of 2.30–2.85 with a Lift Coefficient of 1.78, compatible with the observed data^37^ at mean GR of 2.32, airspeed of 7.51 m s^−1^, indicating that the Flight 1.25 model^13^ can effectively reproduce the gliding mechanics of mammals. A further test was carried out by simulating the glide of the smaller N American squirrel, *Glaucomys sabrinus* (Sciuridae) based on published data^54^ with average AR^67^. The very steep angle of glide and thus angle of attack was simulated by increasing the wing profile drag coefficient to 0.3. The results indicate a glide speed of 7.1 m s^−1^ (best-glide) and GR of 1.8 to 2.1, values that closely match the observed^54^ average glide speed of 7.2 m s^−1^ and GR of 1.98.

Testing the model with the flight of extant bats is more complex as aerodynamic theory suggests that minimum power and, thus, preferred flight speed should increase with mass^21,63^. Bats can modify the shape of their wings^21^, and large bats can adopt higher lift coefficients or modify wingbeat frequency and angle of attack. Test flights in corridors and wind tunnels are also artificial environments that might affect bat speed. The Flight 1.25 model^13^ can reproduce bat as well as bird flight, and using the parameters for extant bats of: wingbeat reduction factor of 0.68, body drag coefficient of 0.25, frontal area coefficient of 1.3, and muscle mass of 9%, simulations were carried out for the bat species *Rousettus aegyptiacus*, *Cynopterus brachyotis* (Pteropodidae), *Glossophaga soricina* (Phyllostomidae) and *Tadarida brasiliensis* (Molossidae; Supplementary Table 4). The measured values of airspeed, with Lift Coefficient (*L*) around the normal flight value of less than 1^30^, can then be compared with speeds predicted by the model, i.e., the range between minimum power airspeed (VmP) and maximum range airspeed (VmR).

The simulated value of wind tunnel speed is slightly lower than the average for *Rousettus aegyptiacus*, a commuting bat^73^; it is also slightly lower for *Glossophaga soricina* and *Cynopterus brachyotis*, while it was clearly lower for *Tadarida brasiliensis*. Thus, VmP as determined by the Flight program^13^ tends to be on the conservative side for airspeeds, but within the observed VmP-to-VmR range of speeds, so the overall model is generally validated, only with some caveat for highly specialized bats, such as *Tadarida brasiliensis*—an open-space, high-altitude flight specialist^73^. The horizontal velocities of pteropodid bats^30^ concentrate around 4.98 m s^−1^, with non-wind tunnel data being slightly mass dependent; thus, simulation values greater than 6 m s^−1^ would indicate airspeeds higher than levels in extant species, putting the animal at risk from collisions.

### Further sensitivity tests

Under the gliding transition theory being tested here, a critical stage in bat evolution is when gliding can potentially be enhanced by flapping, as represented by Model 3, with a limb patagium AR of 2.6 and the enclosed handwing extension giving a total AR of 3.72, this requires lowering the angle of attack such that the animal became a more efficient airfoil at acceptable airspeeds. In the Flight 1.25 program^13^, the effect of ‘flattening’ the glide (i.e., diminishing the glide angle) can be modeled by adopting wing drag coefficients reported for flying squirrels and sugar gliders^74^ of 0.1; 0.15, and 0.2 for approximate angle of attack of 10, 20 and 30 degrees. Fight 1.25^13^ simulations of the Southern flying squirrel (*Glaucomys volans*; Sciuridae) and sugar gliders (*Petaurus breviceps*; Petauridae) show that at a ‘flattened’ glide of ~10 degrees, glide ratio is increased from 2.07 to 2.77, but this gain comes with a cost: airspeed would be 9.4 ms^−1^, greatly increasing potential impact energy. Simulations of the fossil body mass of 32– 48 g (±20% the original mass estimate) at different wing drag coefficients were carried out with the whole animal treated as an airfoil, as per Pennycuick^13^, and wing drag coefficients ranging from 0.1 to 0.3, as shown in Supplementary Table 5. It is stressed that these represent steady-state values and that most gliding animals are capable of changing pitch to increase or modify airspeed as required. The simulations as summarized in Supplementary Table 5 indicate that glide ratio is more sensitive to angle of attack than changes in mass. However, in normodense air and a ‘flattened’ angle of attack of 10 degrees, airspeeds higher than the cutoff value of 6 ms^−1^ are suggested for a body mass superior to 36 g (shaded in Supplementary Table 5). In hyperdense conditions, variable angles of attack over all mass values are within the safer airspeed envelope, allowing glide ratio to be extended.

A further sensitivity test on muscle mass was also carried out for powered flapping, initially testing the same range of mass estimates for Model 3 with the ascribed value of flight muscle of 8% of total body mass. A further test was carried out using a muscle mass of 10%, as used in the evaluation of flight in theropod dinosaurs^29^, although this is higher than the median value for extant bats^63^ and very unlikely in *Onychonycteris finneyi*. Supplementary Table 6 shows that for all the body mass estimates with a muscle mass of 10% vertical lift (climb) was positive, however, airspeeds were greater than 6 ms^−1^ in normodense air. In hyperdense air, however, minimum power airspeeds were below 6 ms^−1^. An increased power/flight muscle mass certainly improves flight, but only in hyperdense air are airspeeds with the safety envelope (<6 m s^−1^).

### Estimation of possible eocene air densities

Composition of palaeo-atmosphere is extremely difficult to determine in any direct way; thus, proxies are typically used to estimate composition, mass and properties of past atmosphere. Traditional models of estimating the mass of these gases from bulk rock calculations, such as Geocarbsulf (GCS)^75^ and related models, assume a constant mass of nitrogen over geologic time. However, the assumption that atmospheric mass should be constant over Earth´s history is not an inherent property of the planet and may not be valid as PATM has both increased and decreased many times in the geological past^76–78^. In fact, analysis of two independent proxies, as outlined below, strongly suggests elevated PATM and, consequently, higher air densities for the Eocene, as anticipated from biological phenomena^23,24^. In the following, air pressure, air density and atmospheric mass are taken to scale at approximately the same values over the variations being considered, hence an increase of, for instance, 30% in standard air density; PATM in bar; and atmospheric mass of 1 atmosphere baseline; are all represented as being equivalent to 1.3 bar.

The first proxy we use is marine versus terrestrial-derived *p*CO_2_. Boron isotopes in foraminifera have been used to record seawater pH and, consequently, the partial pressure of atmospheric CO_2_ in equilibrium with this water^78^. Low pH values of between 7.8 (40 Ma) and 7.6 (53 Ma) have been found for the Eocene^79^, from which *p*CO_2_ of about 1400 ppm (parts per million) has been derived^78^. This technique suffers from some limitations, such as the influence of vital effects^80^ and size of the foraminifera; thus an attempt to resolve the discrepancy between these high CO_2_ levels and terrestrial data suggested a basic calcite-derived CO_2_ level of 800 ppm for the Middle Eocene^81^. Marine nahcolite and boron data also indicate CO_2_ levels at c. 800 ppm in marine environments by ~50 Ma^25,81,82^. In contrast to this, Eocene CO_2_ terrestrial levels from fossil plant-leaf stomata sources in New Zealand and Australia indicate levels closer to 500 ppm for the period covering 38 Ma–53 Ma^83^, which agree with many other global stomatal studies^25,83–90^. The difference in derived Eocene CO_2_ from marine and terrestrial realms has been termed ‘contradictory’^91^, and is also seen in the Miocene, where ‘conditions have been found to be difficult to reconcile with present climate models’^92^. However, the terrestrial (low) and marine (high) controversial values of the Eocene (and Miocene) can be reconciled by introducing atmospheric pressure as a variable. The sea absorbs a large amount of CO_2_ due to the relatively high solubility of this gas in seawater, the dissolved CO_2_ participating in chemical and biological processes while being circulated around the global oceans; i.e., the hydrosphere (or marine) compartment of the biosphere carbon cycle. The solubility of CO_2_ in seawater is expressed by Henry’s Constant (KH) as [mol (kg H_2_O) −1 atm−1]; this is approximately linear over the range 0 °C to 50 °C, and directly proportional to its partial pressure^93^, but highly responsive to air pressure—~55 times greater than that of N_2_ at 20 °C. Hence, CO_2_ solubility in seawater is highly dependent on atmospheric pressure. CO_2_ solubility in seawater, examined from a constant flow of gas mix (2% CO_2_, 98% N_2_) at 1 and 2 bar and over several constant temperatures, indicated that at 25 °C and 1 bar, solubility was 70 (CO_2_ concentration in ppm); at 2 bar, it rose to 550 ppm^94^. Assuming a reasonable linear difference given that CO_2_ solubility in seawater from 0.1 bar to 0.9 bar is almost linear^94^, this gives a gradient of about 48 ppm per 0.1 bar increase. This relation can therefore be used to match the observed stomata or terrestrial-derived and boron or seawater-derived *p*CO_2_ such that:

$$M{{{{{\rm{arine}}}}}}\,{p}{{{{{{\rm{CO}}}}}}}_{2}(800\, {{{{{\rm{ppm}}}}}})={{{{{\rm{Terrestrial}}}}}}\, {p}{{{{{{\rm{CO}}}}}}}_{2}(500\, {{{{{\rm{ppm}}}}}})+{{{{{\rm{P}}}}}}(300\, {{{{{\rm{ppm}}}}}})$$

where *P* approximates the extra density component derived from PATM. Thus, the difference of 300 ppm based on the CO_2_ gradient of 48 ppm per 0.1 bar from data on CO_2_ solubility in seawater^94^ suggests an extra component of 0.63 bar, giving a paleo PATM estimate of c. 1.63 bar for the early Eocene.

The second proxy we use is carbon isotopes in amber. Resins have chemical properties that make them particularly suitable as proxies of environmental changes over geologic time, as these properties have not changed much with plant evolution^28,95^. Thus, for profuse resin producers, it can be assumed that the metabolized CO_2_ was sourced from isotopically undisturbed air that had a δ13C composition approximating a global atmospheric average. An evaluation of the effect of a variable *p*O_2_ on amber isotopes, using experimental work on the isotopic fractionation in C3 plants and partial oxygen pressure, revealed that the fractionation of carbon during photosynthesis was found to increase when *p*O_2_ in the ambient air is higher than modern values, resulting in depleted δ13C plant mass^27^. The opposite effect has also been observed under lower than-modern *p*O_2_ in ambient air^96^. From these observations, a direct relationship is a reasonable assumption for moderate *p*O_2_ levels, provided that major physiological adaptations of plants are not involved^28^. In this empirical model, paleo-*p*O_2_ at the time of resin formation may have been as low as 13% in the Eocene—a value starkly at variance with all versions of the Geocarbsulf and Geocarbsulfor models, which predict similar *p*O_2_ levels for the past 50 Ma in relation to the c. 21% value at present^75^.

Low *p*O_2_ for the Eocene presents some apparent problems. Fire activity is effectively switched off at *p*O_2_ < 16%, but was greatly enhanced at 22%^97^ so it has been suggested that the low *p*O_2_ values derived from amber are incompatible with wildfire data^98^. However, experiments at varying pressures indicate that the *p*O_2_ minimum for fire is a function of the product of *p*O_2_ and PATM^99,100^, such that these low oxygen values would be sufficient for the wildfire propagation seen in the record at higher PATM. These low values are also seen in air trapped in Eocene halite and derived from a revised pyrite proxy indicating that this was a period of air density higher than at present times^101^. Thus, the mass estimates of O_2_ based on the Geocarbsulf model data^26,83^ and on the *p*O_2_ derived from resin / amber data can be reconciled by varying the PATM values (air density) of the resin model such that the O_2_ mol m^-3^ are similar, essentially by increasing the atmospheric mass by the relevant factor. Thus for any given period:

$${{{{{\rm{Palaeo\; PATM}}}}}}=(p{{{{{{\rm{O}}}}}}}_{2}\,{{{{{\rm{GCS}}}}}}/p{{{{{{\rm{O}}}}}}}_{2}\,{{{{{\rm{AMB}}}}}}){{{{{\rm{bar}}}}}}$$

where *p*O_2_ GCS is the value estimated from the Geocarbsulf model (21%) and *p*O_2_ AMB is from amber data (averaged 13% from above sources). These factors suggest estimates of PATM (in bar) of between 1.44 and 1.64 for the Eocene (c. 50 Ma). Recent Eocene amber data^102^ also suggest a sharp drop in PATM during the Eocene to Oligocene Transition, thus at ~50 Ma, possible levels of PATM were between 1.54 and 1.6 bar, suggesting the maximum constrained value tested of 1.6 bar.

### Statistics and reproducibility

All data used are provided in the Supplementary Tables. Aerodynamic results can be reproduced by introducing these data in the freely available aerodynamic model programme Flight 1.25^13^.

### Reporting summary

Further information on research design is available in the Nature Portfolio Reporting Summary linked to this article.

### Supplementary information

Supplementary Information

Reporting Summary

## Data Availability

All data generated for this study are available in the Main Text and the Supplementary Information.
