# Supplementary material for: Palaeoatmosphere facilitates a gliding transition to powered flight in the Eocene bat, Onychonycteris finneyi
Source: Commun Biol. 2024 Mar 26;7:365. doi: 10.1038/s42003-024-06032-9 (PMC10966098; doi:10.1038/s42003-024-06032-9)
Supplement: Supplementary file 1 — Supplementary Information [file 42003_2024_6032_MOESM1_ESM.pdf]

Supplementary Information for:

Palaeoatmosphere facilitates a gliding transition to powered flight in the Eocene bat, *Onychonycteris finneyi*

Norberto P. Giannini, Alan Cannell, Lucila I. Amador, Nancy B. Simmons

Contents

Supplementary Figure 1. Reconstructed aerofoil in all models..2

Supplementary Note 1. On flight speed and collision risk..3

Supplementary Table 1. Flight characteristic of *Onychonycteris finneyi* and Model 4..............4

Supplementary Table 2. Glide features of Models 1-to-4 and the full-winged *Onychonycteris*...5

Supplementary Table 3. Wing parameters of all models..6

Supplementary Table 4. Simulated versus observed flight speeds in selected bats................7

Supplementary Table 5. Variation of Model 3 glide airspeeds..8

Supplementary Table 6. Model 3 flapping airspeeds and climb rates 9

Supplementary Table 7. Numerical value sources for Figure 2......................................10

Supplementary references..11

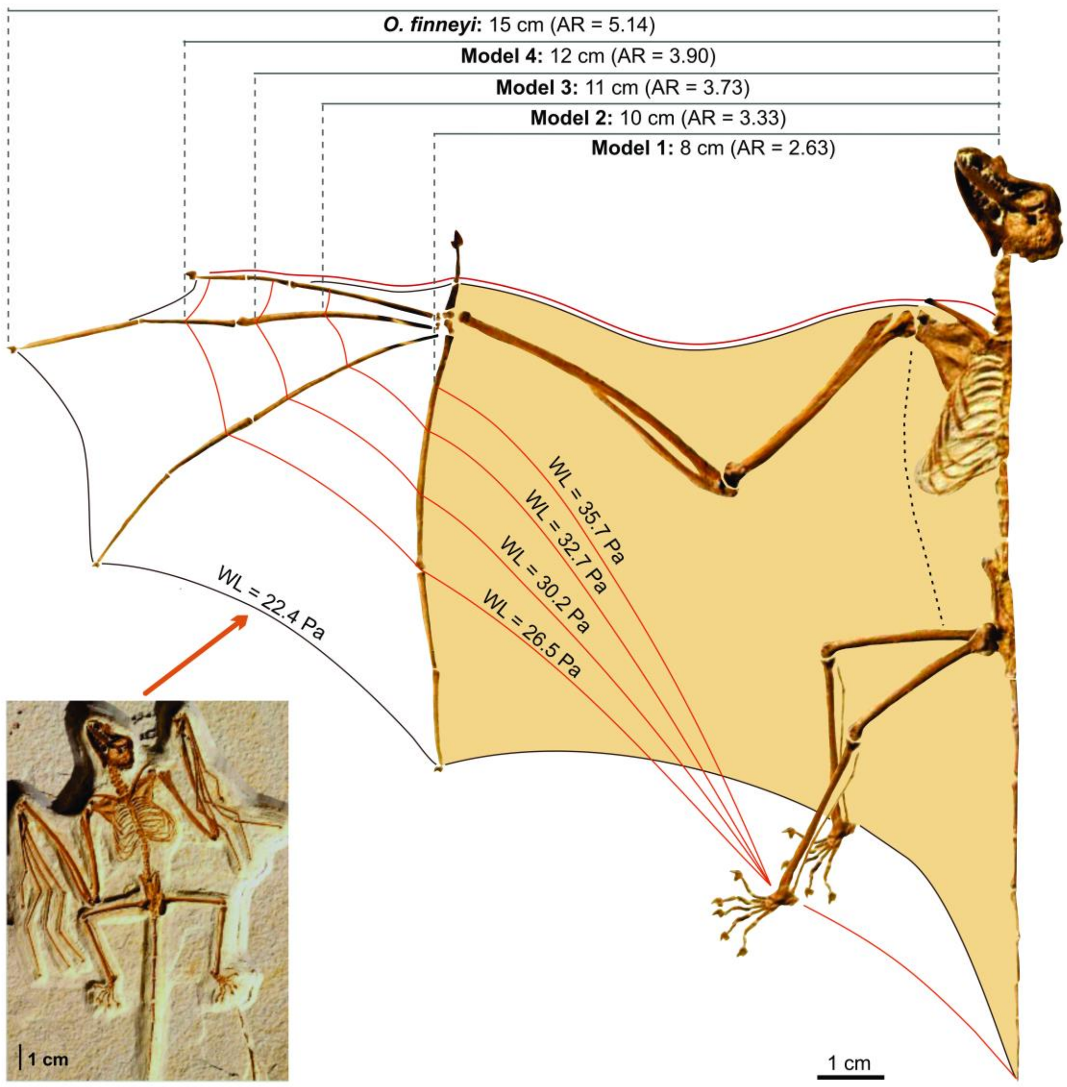

Supplementary Figure 1. Reconstructed aerofoil in *Onychonycteris finneyi* (full contour, modified from ref.[1]) stretching the hindleg as seen in the profile of extant gliders[2]. Models (Gm 1-to-4) are illustrated overlapping in red contour with indicated half wingspan (b/2), aspect ratio (AR) and wing loading (WL). The functional handwing is unshaded and anatomically it includes the dactylopatagium (membrane between digits 2-to-5) and propatagium brevis (membrane between digits 1-to-2). The shading in the wing includes the propatagium longus, plagiopatagium and uro-patagium (the basic gliding *bauplan*) and indicates aerofoil surface without handwing using the hingleg posture unmodified from the fossil. Inset: paratype American Museum of Natural History AMNH 142467.

Supplementary Note 1:

Additional considerations on flight speed and collision risk

Most organisms can withstand impacts at 4.4 m s^{-1} but impacts at greater speeds can be fatal[3]. Mean airspeed for the diurnal migrating moth *Urania fulgens* with a mass of about 0.5 g was found to be about 3.8 m s^{-1} [4] and slightly lower for British Nocturnal moths[5], so it would be expected that an insectivorous bat would be able to fly at close to this prey speed. Fruit bats and bats that forage over large distances can, and would be expected to, have a greater AR, more pointed wingtips and be capable of higher flight speeds[6]. However, Barn Owls search for woodland prey at similar speeds of 4.5 to 9 m s^{-1} – approaching in a silent obligate glide (Audubon.org). Flying squirrels and gliding dracos also have glide speeds of ~6 to 7 ms^{-1} [7].

When faced with an unfamiliar artificial woodland environment, big brown bats (*Eptesicus fuscus*) change their behavior and reduce flight speed to a mean of 2.49 m s^{-1} as compared to 3.01 m s^{-1} in an open space[8]. Similarly, when pigeons have to negotiate a 'forest' of poles, their flight speed is reduced 44.5% from 6.95+0.64 m s^{-1} to 3.86+0.52 m s^{-1} [9]. Both birds and bats thus react to the risks of tree or branch collisions by adopting conservative speeds in woodland environments due to the law of physics in which impact energy and injury risk increases exponentially with collision speed: at a flight speed of 11 m s^{-1} impact energy is 3.4 times that at 6 m s^{-1}. This is confirmed by a recent study[10] that shows the impact damage of birds hitting unseen glass windows at different speeds – the cause of millions of bird deaths annually. Most visible injuries were considered to be life threatening. At 6 to 7 m s^{-1} risk is almost negligible. At 10 m s^{-1} about 50% of collisions result in death.

Supplementary Table 1. Flapping flight Characteristics of Model 4 as compared to the full-winged *Onychonycteris finneyi* considering a reconstruction of body weight at 40 g versus 10% heavier at 44 g, calculated using Flight 1.25[11]. (Shaded values are considered critical)

Patm bar	Min. Power air speed ms^{-1}	climb ms^{-1}	Power W	Work myofibrils J/kg	Wing-beat Hz	Min. Power air speed m/s	climb ms^{-1}	Power W	Work myofibrils J/kg	Wing-beat Hz
**Model 4**	**40g**					**44 g**				
1	6.8	-0.36	0.77	49.9	6.9	7.1	-0.44	0.81	52.8	7.2
1.6	5.4	-0.06	0.61	43.0	5.8	5.6	-0.15	0.71	45.2	6.0
**fossil**	**40 g**					**44 g**				
1	6.1	0.27	0.46	33.1	5.3	6.3	0.23	0.54	34.5	5.5
1.6	4.8	0.34	0.36	29.8	4.4	5	0.31	0.43	31	4.6

Supplementary Table 2. Glide features of Models 1to 4 and the full-winged *Onychonycteris* using Flight 1.25[11], in two contrasting PATM conditions (normodense at 1 bar and hyperdense at 1.6 bar), mass reconstructed at either 40 g or 44 g. Shaded cells indicate values calculated with a wing drag coefficient of 0.1. Turn radius is at 24° bank. The light gray shaded lines are the model runs with lower wing drag due to the lower angle of attack as mentioned. The highlighted (dark gray) values are considered to represent high risk.

**Modelled 40 g specimen**	**PATM bar**	**GR**	**Best Glide Speed $m\ s^{-1}$**	**Sink Rate $m\ s^{-1}$**	**Impact speed $m\ s^{-1}$**	**Turn Radius m**
Model 1	1	2.93	6.4	2.19	6.8	4.85
	1.6	2.93	5	1.71	5.3	3.07
Model 2	1	3.37	5.7	1.69	5.9	4.42
	1.6	3.37	4.5	1.33	4.6	2.73
Model 3	1	3.61	5.3	1.47	5.5	4.01
	1.6	3.61	4.2	1.16	4.4	2.57
Model 4	1	3.72	5	1.34	5.2	3.62
	1.6	3.72	4.1	1.05	4	2.26
Model 3	1	4.6	6.2	1.23	6.4	4.2
	1.6	4.6	5.2	1.13	5.3	2.88
Model 4	1	4.78	5.8	1.21	6	3.62
	1.6	4.78	4.8	1	4.9	2.55
Fossil	1	5.7	5	0.88	5.1	3.07
	1.6	5.7	4.1	0.72	4.2	2.1
**Modelled 44 g specimen**	**PATM bar**	**GR**	**Best Glide Speed $m\ s^{-1}$**	**Sink Rate $m\ s^{-1}$**	**Impact speed $m\ s^{-1}$**	**Turn Radius m**
Model 1	1	2.92	6.6	2.26	7	5.3
	1.6	2.92	5.2	1.78	5.5	3.42
Model 2	1	3.36	6	1.78	6.2	4.85
	1.6	3.36	4.7	1.4	4.9	3.1
Model 3	1	3.6	5.6	1.56	5.8	4.42
	1.6	3.6	4.5	1.32	4.7	3.07
Model 4	1	3.9	5.1	1.4	5.3	4.21
	1.6	3.9	4.1	1.11	4.2	2.4
Model 3	1	4.58	6.5	1.42	6.7	4.62
	1.6	4.58	5.4	1.18	5.6	3.07
Model 4	1	4.75	6.1	1.28	6.2	4.01
	1.6	4.75	5	1.05	5.1	2.73
Fossil	1	5.67	5.2	0.92	5.3	3.42
	1.6	5.67	4.3	0.76	4.4	2.26

Supplementary Table 3. Wing parameters estimated for the full-winged fossil *Onychonycteris finneyi* (averaged between holotype and paratype) and intermediate models 1-to-4.

**Model**	**Complete Wing Area (m^2)**	**Wingspan (m)**	**Aspect Ratio**
Model 1	0.0110	0.17	2.63
Model 2	0.0120	0.20	3.33
Model 3	0.0130	0.22	3.73
Model 4	0.0148	0.24	3.90
Onychonycteris	0.0175	0.30	5.14

Supplementary Table 4. Comparison of simulated flight speeds, Minimum Power (VmP) and Maximum Range (Vmr), in m s^{-1}, as calculated using Flight 1.25[11] with observed airspeed in selected bat species.

**Species**	**Mass kg**	**VmP m s^{-1}**	**Vmr m s^{-1}**	**Maximum observed airspeed m s^{-1}**	**Source**
Rousettus aegyptiacus	0.120	6.7	11.8	9.0	ref. 11
Cynopterus brachyotis	0.032	5.1	9.2	5.5	ref. 12
Glossophaga soricina	0.011	4.5	8.4	7.0	ref. 12
Tadarida brasiliensis	0.012	4.4	7.9	8.3	ref. 12

Supplementary Table 5. Variation of Model 3 glide airspeeds for different angles of attack and body mass (see Main Text). Simulations done using aerodynamic program Flight 1.25[11]. Abbreviations: AoA angle of attack; GR glide ratio; PATM atmospheric pressure. Shaded values are considered critical.

		**AoA in Degrees and GR Airspeeds in m s^{-1}**					
**Mass g**	**PATM bar**	**10**	**GR**	**20**	**GR**	**30**	**GR**
32	1	5,8	4,81	4,9	3,7	4,4	3,12
	1,6	4,6		3,8		3,4	
36	1	6,1	4,79	5,1	3,69	4,6	3,12
	1,6	4,8		4,1		3,6	
40	1	6,4	4,77	5,4	3,68	4,9	3,11
	1,6	5,1		4,3		3,8	
44	1	6,7	4,75	5,7	3,67	5,1	3,11
	1,6	5,3		4,5		4	
48	1	7	4,74	5,9	3,66	5,3	3,1
	1,6	5,5		4,7		4,2	

Supplementary Table 6. Model 3 airspeeds and climb rates at different body mass values and a 10% flight muscle mass. Abbreviation: PATM atmospheric pressure. Shaded values are considered critical.

**Mass g**	**PATM bar**	**Min. Power airspeed m s^{-1}**	**Climb rate m s^{-1}**
32	1	6.40	0,4
	1,6	5.00	0,5
36	1	6.60	0,32
	1,6	5.20	0,46
40	1	6.80	0,23
	1,6	5.40	0,4
44	1	7.10	0,14
	1,6	5.60	0,35
48	1	7.30	0,05
	1,6	570	0,29

Supplementary Table 7. Numerical values represented in the plot of Figure 2 (Main Text). Abbreviations: AR aspect ratio; BM body mass; PATM atmospheric pressure.

Model	AR	BM	Sink (-) or Climb (+) rate (m/s)	
			PATM: 1 bar	PATM: 1.6 bar
1	2.63	40 g	-2.19	-1.71
		44 g	-2.26	-1.78
2	3.33	40 g	-1.69	-1.33
		44 g	-1.78	-1.4
3	3.73	40 g	-1.47	-1.16
		44 g	-1.56	-1.32
4	3.90	40 g	-1.34	-1.05/-0.06
		44 g	-1.4	-1.11
Fossil	5.14	40 g	-0.88/+0.27	-
		44 g	-0.92	-

Supplementary references:

1. Amador L. I., N. B. Simmons, N. P. Giannini, Aerodynamic reconstruction of the primitive fossil bat *Onychonycteris finneyi* (Mammalia: Chiroptera). *Biol. Letters* **15**, 20180857 (2019).
2. Jackson S. M., *Gliding Mammals of the World* (Csiro Publishing, 2012).
3. Vogel S., Living in a physical world VI. Gravity and life in the air. J. Biosciences 31, 13-25 (2006).
4. Dudley, R., Srygley, R.B., Oliveira, E.G., & Devries, P.J. (2002). Flight Speeds, Lipid Reserves, and Predation of the Migratory Neotropical Moth Urania fulgens (Uraniidae)1. Biotropica, 34, 452-458.
5. Hayley Bridgette Clarke Jones. Quantifying dispersal in British noctuid moths. PhD Thesis, University of York, September 2014
6. Norberg U. M., J. M. Rayner, Ecological morphology and flight in bats (Mammalia; Chiroptera): wing adaptations, flight performance, foraging strategy and echolocation. Phil. Trans. Roy. Soc. London B 316, 335-427 (1987).
7. McGuire, J.A., & Dudley, R. (2011). The biology of gliding in flying lizards (genus Draco) and their fossil and extant analogs. Integrative and comparative biology, 51 6, 983-90.
8. Falk B, Jakobsen L, Surlykke A, Moss CF. Bats coordinate sonar and flight behavior as they forage in open and cluttered environments. J Exp Biol. 2014 Dec 15;217(Pt 24):4356-64. doi: 10.1242/jeb.114132. Epub 2014 Nov 13. PMID: 25394632; PMCID: PMC4375838.
9. Lin H-T, Ros IG, Biewener AA. 2014 Through the eyes of a bird: modelling visually guided obstacle flight. J. R. Soc. Interface 11: 20140239. http://dx.doi.org/10.1098/rsif.2014.0239
10. Samuels B, Fenton B, Fernández-Juricic E, MacDougall-Shackleton SA. 2022. Opening the black box of bird-window collisions: passive video recordings in a residential backyard. PeerJ 10:e14604 DOI 10.7717/peerj.14604
11. Pennycuick C. J., *Modelling the Flying Bird* (*Theoretical Ecology Series*) (Elsevier, 2008).
12. Hedenström A., L. C. Johansson, Bat flight: aerodynamics, kinematics and flight morphology. *J Exp Biol.* **218**, 653-63 (2015).
